# Poor Prognosis for Puumala Virus Infections Predicted by Lymphopenia and Dyspnea

**DOI:** 10.3201/eid2905.221625

**Published:** 2023-05

**Authors:** Stefan Hatzl, Florian Posch, Marina Linhofer, Stephan Aberle, Ines Zollner-Schwetz, Florian Krammer, Robert Krause

**Affiliations:** Medical University of Graz, Graz, Austria (S. Hatzl, F. Posch, M. Linhofer, I. Zollner-Schwetz, R. Krause);; Medical University of Vienna, Vienna, Austria (S. Aberle);; Icahn School of Medicine at Mount Sinai, New York, New York, USA (F. Krammer)

**Keywords:** Puumala virus, hantaviruses, viruses, infections, poor prognosis, lymphopenia, dyspnea, Austria, United States

## Abstract

We investigated a prospective cohort of 23 patients who had Puumala virus infection in Austria to determine predictors of infection outcomes. We reviewed routinely available clinical and laboratory parameters collected when patients initially sought care. Low absolute lymphocyte count and dyspnea were parameters associated with a severe course of infection.

Hantaviruses are emerging rodentborne pathogens that cause clinical illness in humans. During the past few decades, hantavirus infection outbreaks increased, demonstrating an emerging problem for healthcare systems (*1*). Human hantavirus infections cause 2 well-defined clinical syndromes: hemorrhagic fever with renal syndrome, caused by Old World hantaviruses originating in Europe and Asia; and hantavirus cardiopulmonary syndrome. Hemorrhagic fever with renal syndrome is characterized by acute renal failure, thrombocytopenia, and mortality rates of 0.1%‒0.4%, and hantavirus cardiopulmonary syndrome is characterized by severe involvement of the respiratory and circulatory systems and case-fatality rates >30% (*1*).

Most cases of infection with hantavirus in Europe are caused by Puumala virus (PUUV) (*2*,*3*). Clinical manifestations of PUUV infection vary from subclinical, mild and moderate to severe, with an urgent need for intensive care treatment. However, biomarkers or clinical parameters for risk stratification of PUUV infection are lacking. In this prospective cohort study, we aimed to clarify the prognostic value of routinely assessable clinical and laboratory values in PUUV infection requiring hospital admission.

## The Study

This study was approved by the institutional review board of the Medical University of Graz (approval no. 33-329 ex 20/21). Written informed consent was obtained from all participants.

We performed a prospective, observational, pilot study, enrolling all consecutive adult patients admitted to the Department of Internal Medicine, Medical University of Graz, Austria, because of clinical suspicion of PUUV infection and detection of PUUV IgM by using point-of-care testing (Reagena POC PUUMALA IgM, https://www.reagena.com). We used a PUUV reverse transcription PCR as a confirmation test as described (*2*). Patient data were uniformly collected as described (*4*). We obtained laboratory, clinical, and radiologic data from our in-house electronic healthcare database system and from handwritten charts and inserted these data into a predefined electronic case report form by using REDCap electronic data capture (*5*,*6*). We defined a severe course of PUUV infection if a patient needed oxygen (<92% blood oxygen saturation while breathing ambient air) or hemodialysis or intensive care unit admission.

We performed statistical analyses by using Stata version 16.1 (StataCorp., https://www.stata.com). We report continuous data as medians (25th–75th percentiles) and summarized categorical data by using absolute frequencies and percentages. We applied rank-sum tests, χ^2^ tests, and Fisher exact tests to investigate the association between 1 continuous variable and 1 categorical variable and between 2 categorical variables. To identify variables associated with a severe course of PUUV infection among the 25 variables that were collected in the presence of multiple testing, we prespecified a Sidák corrected α of association, resulting in p values <0.002 to indicate statistical significance. We used logistic models for univariate and multivariable modeling of PUUV infection severity and assessed the optimal cutoff to separate patients with and without severe course of PUUV infection by using a maximized Youdens index within a receiver operating characteristic analysis. The follow-up time was truncated at 90 days after diagnosis because no events were expected after this time interval. We computed overall survival of the cohort by using Kaplan-Meier estimators.

A total of 23 patients were included in the analysis (Table). Median age at diagnosis was 49 years (25th‒75th percentile 34–59 years), and 6 (23%) patients were women. Five (22%) patients had underlying conditions (Table). Ten (44%) patients reported activities with a predisposition to rodents or rodent excreta. The most frequent symptom at diagnosis was fever, which was observed in 100% of the patients. The median body temperature at diagnosis was 39.5°C (25th–75th percentiles 39.0°C–39.7°C). Most (17/23, 74%) patients had headache and reported concomitant use of analgesics. All 3 patients who had dyspnea came to the hospital because of this primary symptom. Symptom duration was 2 (25th‒75th percentile 1–3) days before admission to the hospital.

**Table:** Baseline characteristics for patients according to clinical course and overall for poor prognosis for Puumala virus infections predicted by lymphopenia and dyspnea*

Variable	Overall, n = 23	Mild course, n = 18	Severe course, n = 5	p value
Demographic				
Age, y	49 [34–59]	51 [35–60]	45 [24–49]	0.087
Female sex	6 (26)	5 (28)	1 (20)	1.000
BMI, kg/m^2^	24.9 [23.6–26.8]	24.8 [23.4–27.0]	25.6 [24.8–26.1]	0.433
Comorbidity				
>1	5 (22)	4 (22)	1 (20)	1.000
Symptoms at diagnosis				
Headache	17 (74)	12 (66)	5 (100)	0.133
Eye pain	2 (9)	2 (11)	0	0.435
Dyspnea	3 (13)	0	3 (60)	0.001
Body temperature	39.5 [39.0–39.7]	39.5 [39.2–39.7]	39.4 [39.0–39.8]	1.000
Diarrhea	3 (13)	3 (17)	0	0.328
Abdominal cramps	3 (13)	2 (11)	1 (20)	0.602
Blurred vision	3 (13)	3 (17)	0	0.328
Time from first symptom to diagnosis	2 [1–3]	2 [1–2]	3 [1.3–3.8]	0.047
Predisposition to contact with rodents	10 (44)	7 (39)	3 (60)	0.040
Laboratory values				
Creatinine, mg/dL	1.2 [1.0–1.8]	1.2 [1.0–1.9]	1.2 [0.8–1.3]	0.456
CRP, mg/L	60 [36–73]	58 [42–63]	73 [24–139]	0.551
LDH, IU	266 [236–311]	266 [236–312]	258 [240–280]	0.654
Bilirubin total, mg/dL	0.5 [0.3–0.8]	0.3 [0.3–07]	0.7 [0.6–0.9]	0.107
Prothrombin time, s	105 [95–118]	108 [105–120]	94 [81–97]	0.012
APTT, s	33 [31–37]	33 [31–36]	36 [35–41]	0.147
Blood counts				
Leukocytes, Gg/L	9.9 [9.9‒11.8]	9.0 [6.6–11.1]	12.2 [9.1–13.9]	0.371
Neutrophils, G/L	6.8 [4.1–10.7]	6.3 [4.0–9.3]	10.7 [7.0–12.1]	0.168
Lymphocytes, G/L	1.0 [0.8–2.4]	1.8 [0.9–2.4]	0.3 [0.2–0.5]	<0.001
Thrombocytes, G/L	73 [50–130]	63 [47–129]	78 [73–129]	0.911
Hemoglobin, mg/dL	14.5 [13.2–15.5]	14.4 [13.2–15.4]	15.5 [13.8–16.4]	0.371
Outcome				
Died	1 (4)	0	1 (20)	0.483
Length of stay in hospital, d	7 [4–12]	6 [3–9]	15 [12–17]	0.048

*Values in brackets are 25th–75th percentiles; values in parentheses are percentages. APPT, activated partial thromboplastin time; BMI, body mass index; CRP, C-reactive protein; G, giga; LDH, lactate dehydrogenase.

A severe course of the PUUV infection was observed in 5 (22%) of 23 patients. For these 5 patients, severe PUUV infection was diagnosed by need for oxygen therapy (n = 5), intensive care unit admission (n = 4), and renal replacement therapy (n = 2). Two of those 5 patients had to be treated with extracorporeal membrane oxygenation. Median length of in-patient hospital stay was 7 (25th–75th percentiles 4–12) days. One patient died from multiorgan failure after PUUV infection, corresponding to a 90-day overall survival rate of 96% (95% CI 73%–99%).

Dyspnea, predisposition to contact with rodents, shortened prothrombin time, and low absolute lymphocyte (ALC) count when patients sought care were associated with severe PUUV infection at the 5% level. However, because of the prespecified α corrected for multiple testing, we identified only dyspnea and a low ALC as major predictors for this outcome. Median ALC levels were 1.8 giga (G)/L (25th–75th percentiles 0.9–2.4 G/L) for patients who had nonsevere PUUV and 0.3 G/L (25th–75th percentiles 0.2–0.5 G/L) for patients who had severe PUUV (p<0.0001) (Figure, panel A).

**Figure:**
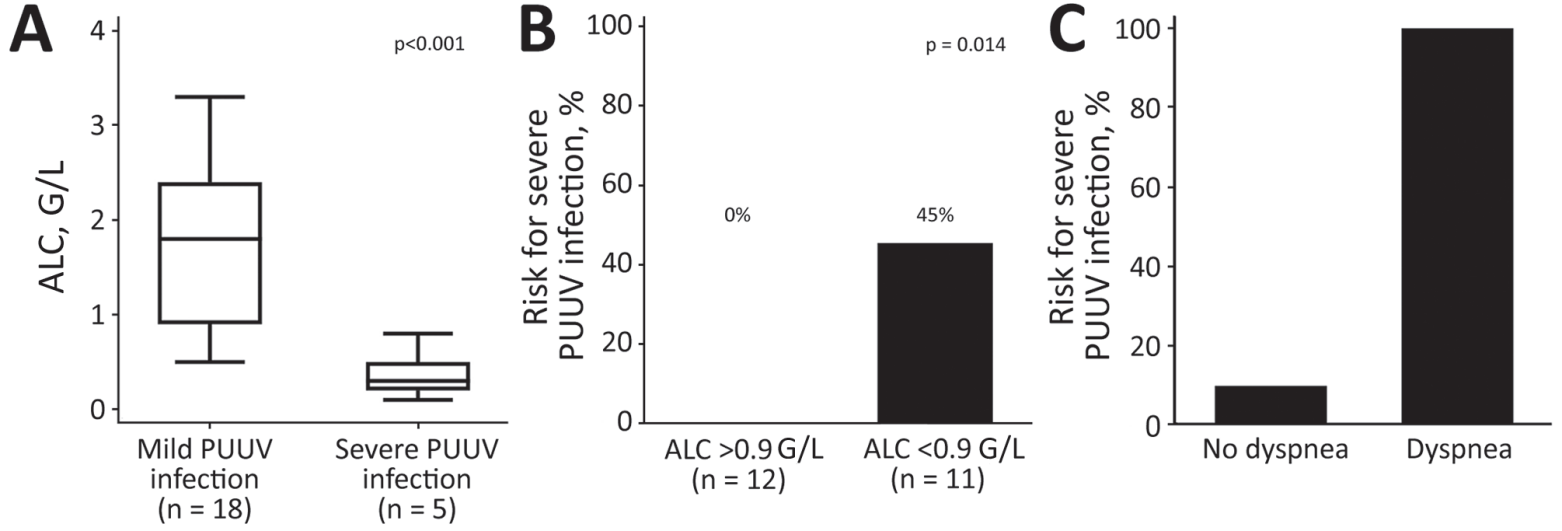
Poor prognosis for PUUV infections predicted by lymphopenia and dyspnea. A) Box plot showing difference in ALC between patients who had a mild clinical course and those who had a severe clinical course, showing that those with lower ALCs were more likely to have severe illness. Horizontal lines within indicate medians, top lines are maximum values, bottom lines are minimum values, and error bars indicate 25th‒75th percentiles. B) Risk for severe course of PUUV infection according to the calculated ALC cutoff of 0.9 g/L, showing that lower ALC predicted increased risk for severe illness. C) Risk for developing severe PUUV infection according to dyspnea at first medical contact, showing that dyspnea predicted increased risk for severe illness. ALC, absolute lymphocyte count; G, giga; PUUV, Puumala virus.

Univariable logistic regression showed that a 0.1 G/L decrease in ALC predicted a 2.3-fold increase in risk for severe PUUV infection (odds ratio 2.28, 95% CI 1.03–5.03; p = 0.042). The area under the receiver operating characteristic curve for ALC for discriminating between patients with and without severe PUUV infection was 0.97, and an ALC cutoff <0.9 G/L was computed for identifying patients at high risk for severe PUUV infection. The risk for severe PUUV infection was 0% for the 12 patients who had an ALC >0.9 G/L and 45% for the 11 patients who had an ALC <0.9 G/L (p = 0.014) (Figure, panel B).

For the 20 patients without dyspnea at initial evaluation, 2 (10%) cases of severe PUUV infection were observed. In contrast, 3/3 (100%) patients with dyspnea at baseline showed development of severe PUUV infection (p = 0.006) (Figure, panel C).

## Conclusions

Risk stratification is crucial for personalized medicine and optimized patient allocation, especially if outpatient management might be considered. Because PUUV has a benign course in most cases, easily assessable predictors of the disease course support clinicians in their decision on patient management (*7**–**9*). We report a small but well-characterized prospective patient cohort, demonstrating lymphopenia and dyspnea at time of first medical contact and diagnosis of PUUV infection as predictors of adverse clinical course.

Most studies focusing on risk stratification in PUUV infection reported kidney injury and renal failure as clinical endpoints, although PUUV infection causes a much wider syndrome, including renal failure, respiratory failure, bleeding events, and circulatory failure (*8*,*10*,*11*). Therefore, we analyzed a composite endpoint consisting of all relevant complications of PUUV infection to support clinical decisions for admission or outpatient care.

A recent review summarized severity biomarkers in PUUV orthohantavirus infection (*12*). However, almost all mentioned biomarkers are difficult to measure, need special laboratory platforms, or are cost-intensive. In our study, we observed that low ALC and dyspnea are easily accessible markers of poor outcomes in an unbiased approach. In our approach, we used all laboratory parameters, signs, and symptoms observed at first medical contact and meticulously corrected for multiplicity. However, the case number of our study was limited, and the parameters should be seen as clinical warning signs of a potential severe clinical course of PUUV infection. The proposed biomarkers need further validation in independent cohorts to prove their utility in a clinical setting.

Our investigated biomarkers were limited to PUUV-infected patients admitted to the hospital, as described in our inclusion strategy. Because only hospitalized patients were included, the observed mortality rate of 4% is slightly higher than rates reported in epidemiologic studies (*1*,*13*).

In summary, we report a prospective cohort of 23 patients who had PUUV infection in an endemic area in central Europe. Our findings indicate that low ALC and dyspnea are parameters associated with a severe course of PUUV infection.
